# Comparison of physics-based deformable registration methods for image-guided neurosurgery

**DOI:** 10.3389/fdgth.2023.1283726

**Published:** 2023-12-08

**Authors:** Nikos Chrisochoides, Yixun Liu, Fotis Drakopoulos, Andriy Kot, Panos Foteinos, Christos Tsolakis, Emmanuel Billias, Olivier Clatz, Nicholas Ayache, Andrey Fedorov, Alex Golby, Peter Black, Ron Kikinis

**Affiliations:** ^1^ Center for Real-Time Computing, Computer Science Department, Old Dominion University, Norfolk, VA, United States; ^2^ Inria, French Research Institute for Digital Science, Sophia Antipolis, Valbonne, France; ^3^ Neuroimaging Analysis Center, Department of Radiology, Harvard Medical School, Boston, MA, United States; ^4^ Image-guided Neurosurgery, Department of Neurosurgery, Harvard Medical School, Boston, MA, United States

**Keywords:** Image-guided neurosurgery, physics-based deformable registration, finite element methods (FEM), high performance computing, mesh generation

## Abstract

This paper compares three finite element-based methods used in a physics-based non-rigid registration approach and reports on the progress made over the last 15 years. Large brain shifts caused by brain tumor removal affect registration accuracy by creating point and element outliers. A combination of approximation- and geometry-based point and element outlier rejection improves the rigid registration error by 2.5 mm and meets the real-time constraints (4 min). In addition, the paper raises several questions and presents two open problems for the robust estimation and improvement of registration error in the presence of outliers due to sparse, noisy, and incomplete data. It concludes with preliminary results on leveraging Quantum Computing, a promising new technology for computationally intensive problems like Feature Detection and Block Matching in addition to finite element solver; all three account for 75% of computing time in deformable registration.

## 1. Introduction

Cancer continues to be a significant cause of death in the USA and worldwide. The number of Americans with brain tumors is about one million, and about 100,000 will receive a primary brain tumor diagnosis in 2023 (1). Neurosurgical resection is a standard and effective treatment for brain tumor patients. Removing as much of the tumor as possible is imperative to ensure the best results while preserving healthy brain structures. This approach can extend the progression time while reducing symptoms and seizures.

One of the main challenges in neurosurgery is identifying critical areas of the brain responsible for essential functions, such as the motor cortex. These areas are unique to each patient and cannot be located with the naked eye. However, medical imaging has proven to be an asset in overcoming this hurdle. Over the past two decades, advancements in image-guided therapy (2) have allowed surgeons to utilize preoperative imaging (3) for neuronavigation. With visualization (4) and quantitative analysis software systems (5), surgeons can safely remove tumors, such as gliomas, from sensitive brain areas. These advancements have significantly improved neurosurgery's safety and success rates.

Before surgery, a combination of anatomical Magnetic Resonance Imaging (MRI) and functional MRI (fMRI) can pinpoint crucial brain areas that affect functions such as vision, speech and language, or motor control. Moreover, Diffusion Tensor Imaging (DTI) can map out white matter tracts that connect to these essential regions and are located near or through the tumor. These imaging techniques are essential in ensuring the precision of the tumor removal procedure.

**Figure 1:**
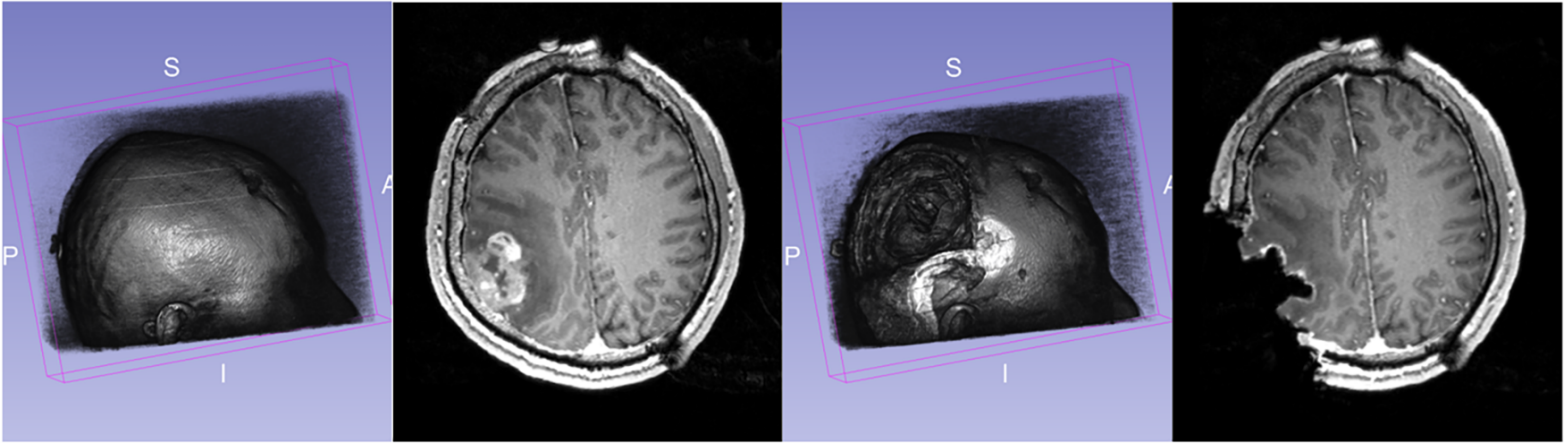
Discrepancies between preoperative and intraoperative MRI before and during neurosurgery: volume rendering and axial view. Preoperative MRI (left) and intraoperative MRI (right) are acquired after a substantial part of the tumor is removed.

During surgery, the opening of the skull and dura causes changes in pressure inside the Intra-Cranial Cavity. Because of this and other factors, such as cerebrospinal ﬂuid drainage and gravity's effect, the brain changes its shape, introducing discrepancies in relation to the pre-operative conﬁguration. The adoption of intraoperative MRI (iMRI) has provided a means for monitoring brain deformation (or brain shift) during surgery (6). Figure 1 depicts the preoperative and interoperative MRI data before and during brain tumor resection. The number of hospitals offering iMRI has grown over the past decade from a handful of research centers to hundreds of clinical sites worldwide (7). Although acquiring fMRI and DTI during surgery may not be feasible, the preoperative images can be aligned with an iMRI through non-rigid registration. The registration results could be applied to preoperative fMRI and DTI, offering more accurate and updated guidance to the neurosurgeon (8). Deformable transformation use on fMRI and DTI is beyond the scope of this paper. This study evaluates deformable registration accuracy between pre-op MRI and intra-op MRI.

## 2. Background

Image registration, in general, is concerned with the spatial alignment of corresponding features in two or more images. During image registration, a spatial transformation is applied to one image (called ﬂoating) to be brought into alignment with the fixed or target image, which is used as a reference position of the object (patient's brain). In the registration process, the ﬂoating image corresponding to the pre-operative MRI is aligned with the patient's position using Rigid Registration (RR), a global transformation. Then, physic-based *non-rigid* registration (PBNRR) uses spatially varying (i.e., local) transformation to account for brain shift, which drastically varies in different brain locations (9). Image registration algorithms generally optimize specific similarity criteria between the ﬁxed and ﬂoating image under varying spatial transformation parameters. The computational complexity of this optimization depends on the number of parameters that describe the transformation.

**Figure 2:**
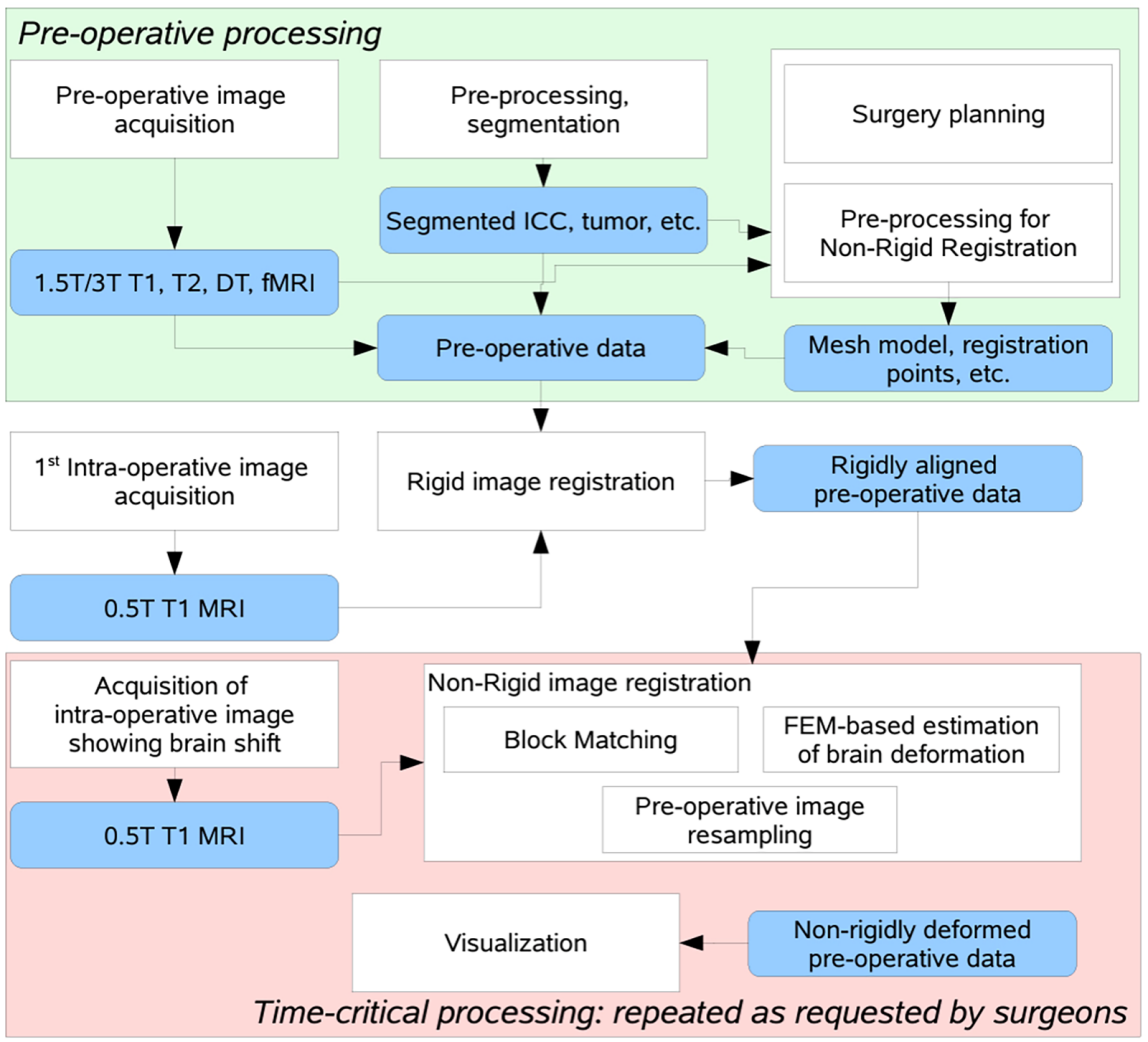
Flow diagram of the NRR process used in BWH during a clinical study, after decoupling the non-rigid registration software to manage fault-tolerance of the distributed computation process (10).

Figure 2 depicts a flowchart with all steps and software modules for pre- and intra-operative image processing for image-guided neurosurgery at Brigham and Women's Hospital (BWH) in Boston, MA. The intra-operative images were 0.5 T iMRI (11) and acquired during surgery at BWH, since then the facility is upgraded and currently using more advanced intraoperative devices (12). The patient-specific Finite Element (FE) model, selection of registration points, and non-rigid registration took place remotely at the Center for Real-Time Computing (CRTC) in Virginia using a midsize High-Performance Computing (HPC) cluster of workstations (10).

We first introduce Clatz et al.'s non-rigid registration technique from 2005. Then, we delve into two enhancements that aim to increase its precision when working with sizable brain tumors. The focus is on the elimination of outliers of both points and elements. Outliers emerge from tissue removal in the case of large brain tumors, described in detail by Liu et al. (13) and Drakopoulos et al. (14). We won't delve into previous research by other groups, in particular reviews or comparisons like Sotiras et al. (15), who conducted a comprehensive survey and taxonomy of NRR methods, and Frisken et al. (16), who presented a clinically insightful review at the B-Splines and FE-based methods. Finally, this comparison is meant to complement a companion review of HPC software implementation-related aspects for the same methods, and it will appear in Chrisochoides et al. (17).

## 3. Physics-based non-rigid registration

The specific NRR method was initially developed in INRIA, France, by Clatz et al. (9) and is implemented as open-source software by the CRTC group in Virginia, USA (18). It is designed for registering high-resolution pre-operative data with iMRI—the NRR process takes place in two phases: preoperative and intra-operative. The intra-operative computation is initiated when a scan shows the shift of the brain. The basic idea of the registration method is to estimate the *sparse deformation ﬁeld* that matches “similar” locations in the pre-operative and iMRI and then use a biomechanical model for brain deformation to discard unrealistic displacements so that it can derive a *dense deformation ﬁeld* that deﬁnes a transformation for each point in the image space.

Sparse displacement vectors are obtained at the selected points in the image, where the intensity variability in the surrounding region exceeds a certain threshold. Such *registration (or feature)* points can be identiﬁed before the time-critical part of the computation in the ﬂoating (pre-operative) image. Once the reference (intra-operative) scan is available, the deformation vector is estimated at each of the selected points utilizing block matching (9), where fixed-size rectangular regions (blocks) centered at the registration points are identiﬁed in the ﬂoating image. Given such a block, the method selects a search region (window) in the reference (or fixed) image. At the registration point, the vector of the sparse deformation field is defined by the block's displacement, which produces the most significant similarity between the image intensities in the block and the overlapping section of the window. The normalized cross-correlation similarity metric is used.

It is worth noticing the high computational complexity of the block-matching procedure. Considering the sizes of three-dimensional block and window are deﬁned in pixels as *B *= {*Bx*, *By*, *Bz*} and *W *= {*Wx*, *Wy*, *Wz*}, then the bound on the number of operations is *O*(*BxByBz *× *WxWyWz*), for a single registration point.

The registration is an energy minimization problem (9). One seeks the balance between the external forces, proportional to the sparse displacements, and the internal forces of the mesh resisting deformation:

(1) $$[K\;+\;H^{T}\mathrm{SH}]U\;=\;H^{T}\mathrm{SD}$$

where *K* is the mesh stiffness matrix, *H* is the linear interpolation matrix form the matches to the displacements at mesh vertices, *S* is the block matching stiffness matrix (matches with higher conﬁdence are assigned higher weights), *D* is the vector for the block displacements, and *U* is the unknown displacement vector for mesh vertices. The stiffness matrix, *K*, is calculated based on the assumed physical properties of the brain tissue elastic modulus *E* and Poisson ratio *ν*. This formulation can tolerate some outliers but suffers from a systematic error concerning the correctly estimated displacements. Alternately, one can use approximation to compute the locations of vertices, which would minimize the error concerning the block matches:

(2) $$\arg\underset{U}{\min}(HU-D{)}^{T}S(HU-D)$$

However, this formulation would also minimize displacement error regarding outlier measurements, which one would like to eliminate from the set of block displacements. A robust iterative approach combines approximation and interpolation. Gradual convergence to the interpolation solution is achieved using the external force *F* added to the formulation (1) to slowly relax the internal mesh stress:

(3) $$[K\;+\;H^{T}\mathrm{SH}]U\;=\;H^{T}\mathrm{SD}\;+\;F$$

Rejection of the outlier matches is done iteratively, with a user-deﬁned total percentage of matches to be discarded, *f*_R_, and the number of rejection iterations, *n*_R_, as follows:

1: **INPUT**: nR, fR
2: **for** i = 0 to n_R_ **do**
3:   F_i_ ⇐ KU_i_
4:   U_i + 1_ ⇐ [K + H^T^ SH]^−1^[H^T^ SD + F_i_]
5:   for all blocks k **do**
6:     compute error function *ξ*_k_
7:   **end for**
8:   reject f_R_/n_R_ blocks with the highest error
9:   re-compute S, H, D
10:  **end for**
11: **repeat**
12:  F_i_ ⇐ KU_i_
13:  U_i+1_ ⇐ [K + H^T^ SH]^−1^[H^T^ SD + F_i_]
14: **until** convergence

The force, *F*, is computed at each iteration to balance the internal force of the mesh, *KU_i_*. The error, *ξ*_k_, measures the difference between the block displacement approximated from the current deformed mesh and the matching target for the *k*th block. The user-deﬁned percentage of the displacements with the highest *ξ*_k_ values is rejected. This method converges to the formulation in (2) and is simultaneously tolerant to most point outliers due to faulty matching. However, large brain shifts due to tumor resection with drastic changes in the geometry, the fixed (iMRI) creates element outliers that need to be considered and we address in Section 4.

## 4. Nested expectation maximization method

This section summarizes an extension of the PBNRR by identifying and removing additional type (element) outliers due to tissue resection using the Nested Expectation and Maximization method, referred to as NEMNRR (13). The NEMNRR method formulates the registration as a three-variable (point correspondence, deformation field, and resection region) functional minimization problem, in which point correspondence is represented by a fuzzy assign matrix; the deformation field is represented by a piece-wise linear function regularized by the strain energy as in PBNRR (9), but this time extends the model from a single homogenous tissue to a heterogeneous multi-tissue based biomechanical model. A Nested Expectation and Maximization framework is developed to resolve these three variables simultaneously (13).

The NEMNRR method extends the cost function used in Clatz et al. (9) to:

(4) $$\begin{array}{c} J(U,C,M_{Rem})\;=\sum_{e_{i}\in M\;\setminus \;M_{Rem}}U^{T}K_{e_{i}}U+\\ \;\;\lambda_{1}\sum_{s_{i}\in M\;\setminus \;M_{Rem}}{\left(HU-D(C)\right)}^{T}W(HU-D(C))\\ +\lambda_{2}\sum_{e_{i}\in M_{Rem}}V_{e_{i}} \end{array}$$

where the continuous domain *Ω* (brain image) is discretized as a multi-tissue mesh *M* using the method presented in Liu et al. (19, 20) on a multi-label image segmented from the pre-operative MRI. *M_Rem_* is the removed mesh approximating the resection region $\Omega^{\prime }$. $K_{e_{i}}$ is the element stiffness matrix of element *e_i_*. Each element is associated with a tissue label, which determines the elastic parameters to build the element stiffness matrix. The first term of Equation (4) approximates the strain energy as in Clatz et al. (9), and the third term approximates the volume of the resection region, in which $V_{e_{i}}$ is the volume of element *e_i_*. In the second term, the entries of the vector *D* are defined as

$$d_{i}(c_{ij})=s_{i}-\underset{t_{j}\in \Omega_{R}}{\sum }c_{ij}t_{j},\;\forall s_{i}\in M\setminus M_{Rem}.$$

**Figure 3:**
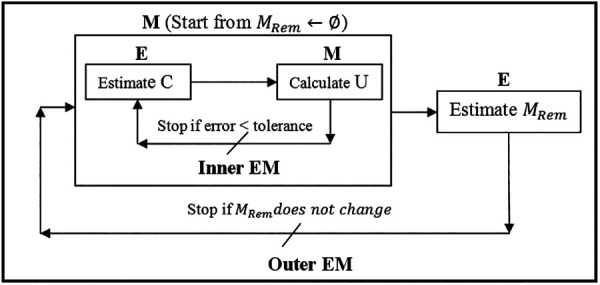
Nested expectation and maximization framework. This figure adopted from Liu et al. (13), Figure 3.

Considering the registration problem in the Expectation and Maximization (EM) context (21), cost function (4), from the probability (Bayesian) point of view, defines the likelihood function, in which the unknown (model parameter) is the displacement vector *U*, and the missing data are the correspondence *C* and the resection region *M_Rem_*. Assuming *M_Rem_* is known, the more accurate the estimate of *C*, the more accurate the estimate of *U*, and vice versa. EM algorithm is very efficient for this kind of circular dependence problem, so one employs EM to solve *U* and *C* under a specified *M_Rem_*. To resolve *M_Rem_*, one can treat *U* and *C* as an approximately known pair *U*, *C*. *M_Rem_* is approximated by a collection of tetrahedra located in a region of the model, which corresponds to the resection region in the intraoperative MRI. *M_Rem_* is initialized to ∅ and updated at each iteration of the outer EM. The outer EM stops if all the tetrahedra contained in the resection region are collected, as shown in Figure 3.

**Figure 4:**
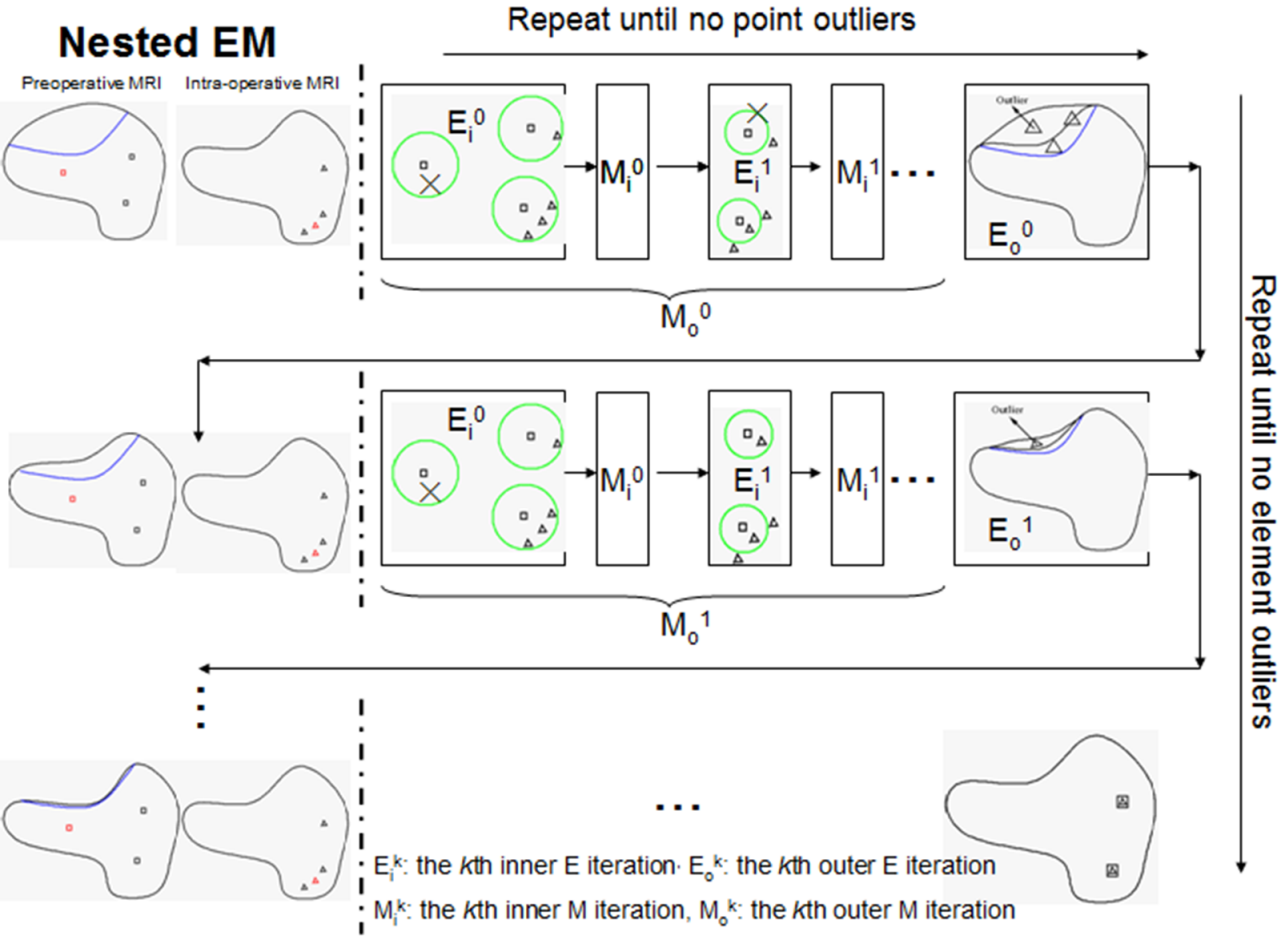
Illustration of nested expectation and maximization strategy. Row: inner EM, Column: outer EM. This figure adopted from Liu et al. (13), Figure 4.

The resection region is difficult to identify in the intra-operative MRI, so a simple threshold segmentation method is used. We cannot determine if a tetrahedron is an outlier based solely on its position. It might be in the background image (BGI) instead of the resection region. To ensure the element outlier rejection algorithm is robust, we use the fact that the resection region is made up of tetrahedra that not only fall in the BGI of intra-operative MRI but also connect and form a maximal connected submesh. The outliers are collected iteratively, with additional outliers added into *M_Rem_* if they fall in the BGI and connect with the maximal simply connected submesh identified in the previous iteration. We demonstrate the NEMNRR strategy in Figure 4, with the inner EM iterating horizontally and the outer EM iterating vertically.

**Figure 5:**
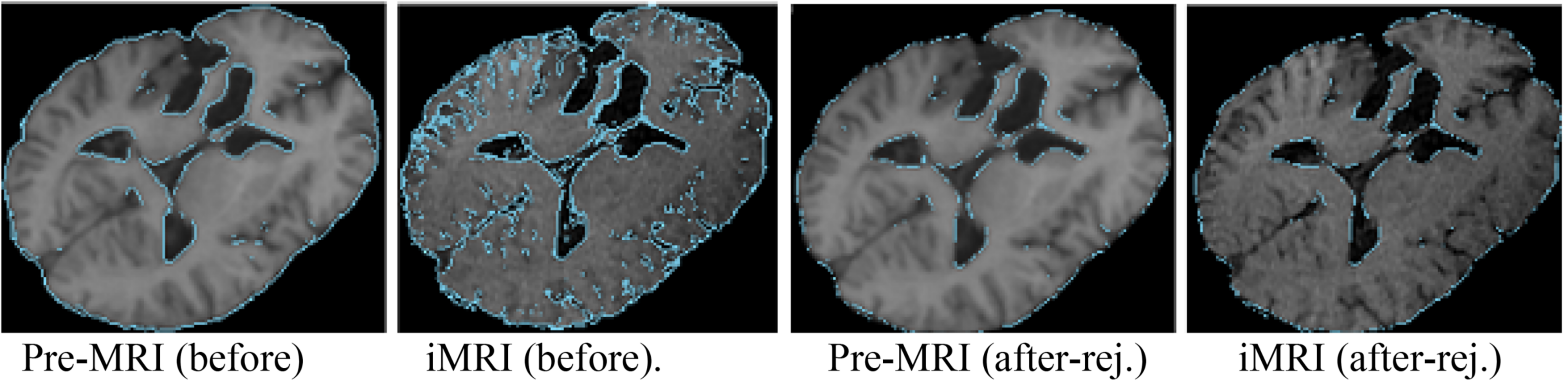
Point outlier rejection. Two left-most pre-op and iMRI depict (in blue) all edges detected before rejecting outliers, while the right-most figures depict the remaining edge points after outlier rejection.

NEMNRR addressed a fundamental challenge in PBNRR: “pre-operative landmarks near the tumor fail to correspond to iMRI landmarks”. The crux of the idea is to use the NEM method to resolve the deformation field with missing correspondence, specifically in the resection region. *This has many implications; one is to compute the registration error more accurately than Hausdorff* Distance *(HD) when correspondence is unknown.* Like the PBNRR, the NEMNRR uses the strain energy of the biomechanical model to regularize the solution. Figure 5 shows the results of point outlier rejection produced by NEMNRR; compared to the edges before outlier rejection, most point outliers are removed from pre-MRI and iMRI after outlier rejection.

## 5. Comparison of outlier rejection schemes

In Sections 5.1, 5.2, we compare two approximation-based outlier rejection methods. Then, in Section 5.3, we compare them with a geometry-based method using 9 cases from earlier studies.

### 5.1. PBNRR rejection scheme vs. rigid registration

First, we compare the PBNRR outlier rejection scheme against state-of-the-art Rigid Registration and B-Splines interpolation schemes with no rejection of outliers. We use five cases from NCIGT (22); they were first presented by Archip et al. (8) with additional analysis by Liu et al. (23). Table 1 lists the patient information, including the gender, tumor location, and histopathology. They are revisited for completeness and reviewed regarding the impact of the outlier rejection scheme on registration accuracy and execution time.

**Table 1:** Patient information from Liu et al. (23).

Case #	Gender	Tumor location	Histopathology
1	F	R occipital	Anaplastic oligodendroglioma WHO III/IV
2	F	L posterior temporal	Glioblastoma WHO IV
3	N/A	R frontal	Oligodendroglioma WHO II/IV
4	N/A	R occipital	N/A
5	F	R frontal	Oligoastrocytoma WHO II/IV

The PBNRR (9) and its implementation in Liu et al. (18, 23) uses a homogeneous model, i.e., a single mesh in the FE model. As reported in Table 2, the execution time is about a minute, and with an average single heterogeneous HPC workstation (with a moderate number of 20 cores and a single GPU, the registration time can be reduced to less than a minute. See Liu et al. (18) for a detailed analysis.

**Table 2:** The quantitative results for the 5 cases are obtained by running the PBNRR on a single homogenous mesh (using 8 threads).

Case	HD before PBNRR (mm)	HD after PBNRR (mm)	HD improvement	Number of registration points	Num rejected outliers	Running time (sec)
1	25.980	20.099	0.226	69,244	**17,310**	57.64
2	9.110	4.690	0.485	76,821	**19,200**	65.88
3	9.433	5.385	0.429	68,745	**17,180**	54.26
4	9.695	7.000	0.278	84,445	**21,110**	67.94
5	6.708	4.123	0.385	68,225	**17,050**	54.92
Avr.	12.18	8.25	0.36	73,496	**18,370**	**60**.**12**

The parameters for all cases are Block radius: [1,1,1], Window radius: [5,5,5], Selection fraction: 0.05, Rejection fraction: 0.25, Num of outlier rejection steps: 10, Num of approximation steps: 10. This table adopted form Insight Journal version of Liu et al. (23).

Bold fonts underline the importance of those points, i.e., removed outliers.

Table 2 indicates that out of about 73 K registration points, the PBNRR rejects about 18 K as outliers. A detailed study of 30 cases by Drakopoulos et al. (24) indicates this leads to a moderate (about 1.5 mm) improvement over the accuracy of the rigid registration and not a clear advantage over the B-Splines approximation scheme implemented in ITK and Slicer3D when accuracy is measured in terms of HD. A recent evaluation (24) from expert neurosurgeons (using specific brain landmarks) indicates that the accuracy of the PBNRR rejection scheme improves the max (and mean) average accuracy to 6.8 (and 3.4) mm from to 8.9 (and 5.6) mm 8.3 (and 4.4) mm by Rigid Registration^1^ and B-Splines^2^, respectively. Overall, the PBNRR^3^ outlier rejection scheme improves the registration accuracy between 1 mm and 2 mm from two state-of-the-art rigid registration schemes.

The end-to-end execution time for registering preoperative to intraoperative images for all 30 cases. Rigid registration, B-Spline, and PBNRR required, on average, 0.84, 8.98, and 0.83 min, respectively (including I/O). The B-Spline method (with comparable accuracy) is the most computationally intensive, requiring more than 8 min in 17 out of 30 cases (24). A different set of B-Spline parameters, such as a smaller sampling percentage, a smaller number of histogram bins, or a coarser grid (than the 15 × 15 × 15 grid used in this study), could improve B-Spline performance at the cost of accuracy.

### 5.2. NEMNRR vs. PBNRR rejection scheme

To compare the two outlier rejection schemes between the NEMNRR and PBNRR, we use three cases from NCIGT (22) and two additional cases from Huashan Hospital (HH) with very large brain shifts. Table 3 lists (the first case from Table 1 and the remaining four cases from an earlier study by Liu et al. (13) the patient information such as gender, tumor location, and histopathology. The thickness slice varies between 1 mm, 1.3 mm, and 2 mm for pre-op MRIs and 1 mm, 2 mm, and 2.5 mm for iMRI. The matrix varies even more, a detailed description is presented in Drakopoulos et al. (24).

**Table 3:** Patient information of five cases for the comparison of PBNRR vs. NEMNRR.

Case	Gender	Tumor location	Histopathology
1 (5, T1, PR)	F	R frontal	Oligoastrocytoma WHO II/IV
2 (9, L, N/A)	F	L Parietal	Glioblastoma multiforme (WHO IV)
3 (10, L, BS)	M	L frontal	Glioblastoma multiforme (WHO IV)
4 (11, L, PR)	M	R temporal	Metastases
5 (12, L, TR)	F	L posterior temporal	Oligodendroglioma WHO II

The case number used in Table 1 (above) and in Liu et al. (13) is in parathesis and denoted by T1 and L, respectively.

Given that NEMNRR is designed to improve registration accuracy using a multi-tissue FEM model, we employ the same multi-tissue mesh in both methods to measure the influence of the outlier rejection scheme on the registration. We build a simple two-tissue mesh (ventricle + the rest of the brain) to minimize the influence of the discrepancy of the geometry and topology between single mesh and multi-tissue mesh. In the homogeneous model, we use Young's modulus = 3,000 Pa, Poisson's ratio = 0.45 for all tetrahedra, and in the heterogeneous model, we replace Young's modulus with 10 Pa and Poisson's ratio with 0.1 for the ventricle (25).

We have seen that, on average, the PBNRR rejects about 18 K outliers out of 73.5 K registration points (approximately 24%) and takes about a minute to complete the registration for an FE-mesh with about 40 K to 50 K elements. Table 4 indicates that for similar cases (see Table 3), the NEMNRR removes 48 K outliers out of 170 K registration points (approximately (28%) at quite a *high cost; NEMNRR takes about six times longer to complete the registration*.

**Table 4:** Selective parameters for NEMNRR and PBNRR related to outlier rejection and execution time.

Case	Multi-tissue mesh		Num. of registration points in the pre-op MRI	Num. of registration points in the iMRI	NEMNRR	PB NRR
	Num. of nodes	Num. of elements			Time	Time
1	48,789	9,322	176,265 (44,703)	168,447 (55,225)	289.8	N/A
2	56,430	10,712	152,798 (50,460)	166,150 (51,723)	678.16	97.6
3	58,558	11,090	247,832 (54,604)	86,720 (41,554)	846.63	101.4
4	80,764	14,893	133,182 (38,350)	147,576 (38,799)	543.42	101.3
5	32,804	6,511	140,598 (53,190)	113,383 (50,359)	548.31	71.2
Avr.	55,469	10,505	**170,135** **(48,261)**	**136,455** (**47,535)**	581.3	92.9

The numbers listed in the column of “Canny” are the edges detected by the Canny edge detector before (and in parathesis after) outlier rejection with NEMNRR. The time for both NEMNRR and PBNRR is in seconds. The bottom row depicts the average of all 5 cases.

Bold fonts underline the importance of those points and removed outliers.

However, NEMNRR increases the accuracy by 9.92 mm on average at the registration points (i.e., the evaluation is performed on Canny edge points) and 2.40 mm when the evaluation is performed on the tumor or resection margin, depending on the case of brain shift or resection. All measurements are based on HD. In 2014, Liu et al. thoroughly compared PBNRR and NEMNRR.

To evaluate the accuracy, we rejected outlier registration points in pre-MRI and iMRI and calculated the HD before registration. Also, we rejected outlier registration points in iMRI and warped pre-MRI to calculate the HD after registration. The tumor boundaries in pre-MRI and iMRI are delineated to calculate the HD for brain shift cases. In each resection case, we choose the pre-MRI slice, in which the tumor is completely intra-operatively resected, so the margin corresponding to the resection margin of iMRI can be identified using the tumor boundary. The resection margin is delineated in iMRI, and directed HD is used for evaluation.

Using HD, the data in Table 5 suggest that NEMNRR and PBNRR do not perform well in the first case, a Partial Resection (PR). In the second case and around the tumor, it appears to be an improvement to about 1 mm. The remaining cases appear to have improved substantially. However, the evaluation results on edges and resection margins must be more consistent within the 1 mm tolerance. This is an area that needs to be studied further. Table 5 indicates that the NEMNRR multi-tissue (Single) reduces the error of Rigid Registration at the registration points from 14.10 mm to 2.5 (2.9) mm, but the evaluation on the resection margin shows the error is reduced only from 12.08 mm to 5.3 (5.6) mm. The reason for this is most likely that the detected edges, although well-aligned, are too far away from the tumor and the resection region and, thus, ineffective in driving the model to estimate the deformation around the resection margin. A larger number of cases (25) were analyzed and compared with both NEMNRR and PBNRR (13), indicating that the mean plus/minus standard deviation for HD between the pre-MRI and iMRI for RR, PBNRR, and NEMNRR is 17.48+/−0.2, 12.7+/−5.4, and 9.8+/−4.7, respectively.

**Table 5:** Quantitative evaluation and comparison for 5 cases.

Case	Rigid		NEMNRR (Single)		NEMNRR (Multi)		PBNRR (Multi)	
	Canny	Tumor	Canny	Tumor	Canny	Tumor	Canny	Tumor
1	13.30	13.01	1.88	11.09	1.85	11.0	N/A	12.0
2	14.28	12.12	3.81	4.32	2.96	4.15	5.98	5.83
3	13.64	10.82	3.96	4.71	3.66	4.63	15.26	16.61
4	13.60	10.77	2.19	5.07	2.14	4.71	17.95	10.08
5	15.26	17.32	2.95	3.0	2.18	2.24	13.40	11.97
Aver.	14.01	12.08	2.9	5.6	2.5	5.3	13.1	11.2

“Single” and “Multi”-denote the single-tissue homogenous and multi-tissue heterogenous model. “Canny” denotes the evaluation performed at the registration points identified by the Canny edge detector, and “Tumor” denotes the evaluation performed on the Tumor or resection margin, depending on whether the case is brain shift or resection.

### 5.3. Comparison with a geometric scheme

In Drakopoulos et al. (14, 24), we used geometric means to remove outliers and attempt to improve the registration error for large tumor resections while staying within the time constraints imposed for clinical use (i.e., completion time 3–4 min). The Adaptive Non-Rigid Registration (ANRR) method gradually adjusts the mesh for the FEM model to an incrementally warped segmented iMRI as opposed to NEMNRR that iteratively rejects feature and element outliers derived from a single (original) segmented iMRI. The idea of the geometric approach is to remove slivers and potentially negative volume elements resulting from large deformation fields (sometimes larger than the size of the elements) computed by block matching. This is achieved through an incremental approximation to reach the end goal. The ANRR method improves the accuracy of the model by improving the accuracy of the basic numerical calculations involved at the cost of increasing (potentially) the overhead for the mesh generation step and substantially increasing the computational cost of the linear solver several times. However, even with a single HPC node (DELL workstation with 12 Intel Xeon X5690@3.47 GHz CPU cores and 96 GB of RAM), the *ANRR execution time on average is less than two minutes* (26)*, which is within the time constraints of the procedure in the operating room*.

Table 6 indicates that a large fraction (about 60%) of time is spent in the parallel FEM Solver module, which includes assembling the system matrices and rejecting the feature (or point) outliers. The differences between the NEMNRR and ANRR are: (1) using different mesh generation methods and (2) treatment of element outlier rejection. In the case of ANRR, we used a Delaunay-based method presented by Foteinos et al. (27), while in NEMNRR, we used the BCC-based method presented by Liu et al. (20). As indicated in the evaluation of both meshing methods in Foteinos and Liu et al. (19, 28), the Delaunay-based method is 15 times faster than the BCC-based method (evaluated on the same set of cases and forced to achieve the same fidelity). However, the BCC-based method is about twice as effective (evaluated in terms of the convergence rate in the FEM-solver) than the Delaunay-based method.

**Table 6:** Profile of the ANRR modules based on total (end-to-end) execution time (in seconds) and relative percentage (%) for each module and adapted from Table 4 (14).

Module name	Time	% of total time
Parallel feature selection	4.38	7.93
Parallel mesh generation	2.58	4.67
Parallel JCG	3.51	6.36
Parallel image def. field update	2.32	4.20
Warped pre-op seg. correction	1.11	2.01
Image def. field correction	2.62	4.74
Parallel FEM solver	33.39	60.58
Parallel block matching	3.77	6.83
Parallel image warping	1.50	2.71
Total	55.18	100

The geometry-based treatment of element outliers (implemented with Parallel Feature Selection, Image Deformation Filed Update and Correction, and Warped pre-op Segmentation Correction presented in Drakopoulos et al. (14) along the real-time I2M conversion technologies like the Delaunay-based method presented in Foteinos et al. (27) to a degree addressed the computational slow-down of NEMNRR (about six times slower compared to PBNRR). It is worth noticing that the role of real-time I2M conversion is not because one needs large FE-meshes for this application, but the requirement is *high fidelity and good quality meshes to be generated quickly*.

Table 7 compares the PBNRR, NEMNRR, and ANRR (with the parameters for all three methods described in Table 9, Appendix I) with two publicly available registration methods: RR and B-Spline (with the parameters for both methods described in Table 10, Appendix I). This time, the comparison is based on two groups of (independent) experts from Europe [AHEPA Hospital in Greece and the results appeared in Drakopoulos et al. (24)] and Asia [Huashan Hospital in China and the results appeared in Liu et al. (13)]. This Table presents each case's minimum, maximum, and mean errors. The assessment confirms that a combination of the Clatz et al. point outlier rejection scheme with the removal of element outliers by alternating PBNRR approximation with remeshing can improve the accuracy of the registration: from an average max (mean) error of 8.4 (4.3) mm achieved by PBNRR to 6.5 (3.2) mm for ANRR as opposed to 7.7 (3.6) mm for NEMNRR.

**Table 7:** Quantitative registration results used six anatomical landmarks for 30 (24) and 25 (13) cases.

Method	Average min error (mm)	Average max error (mm)	Average mean error (mm)
RR	3.19	8.90	5.60
BSPLINE	2.15	8.29	4.40
PBNRR	1.66	8.45	4.36
NEMNRR	1.36	7.79	3.69
ANRR	1.03	6.59	3.22

The average minimum, maximum, and mean errors are computed over thirty for ANRR, NEMNRR, rigid registration (RR), and PBNRR, depicting the average errors of PBNRR, respectively.

To summarize, it is important to note that neither the HD metric nor expert evaluation can be reliably reproduced due to a lack of robustness, and the possibility of human error in expert evaluation. Therefore, combining the two approaches (as we have observed that human error can be caught and corrected using HD results) is the safest way forward until the research community develops more robust and automatic metrics to measure registration accuracy.

## 6. Open problems

### 6.1. Problem I: non-rigid registration error estimation

The evaluation methodology of the analysis we presented in this paper used two methods: (1) expert evaluation (see Table 7), but prone to human errors, and (2) automatic method (30) relying on HD to evaluate the registration accuracy because it is fast and does not require manual intervention (see Tables 2, 5). The automatic method relies on Canny edge detection (31) to compute two-point sets. The first point set is computed from the preoperative volume and then transformed (using the deformation field computed by each registration method) from the preoperative to the intraoperative space. The second point set is computed from the intraoperative volume. An HD metric (32) was employed to calculate the degree of displacement between the two-point sets. This approach helps compare the impact of the different approximation schemes. However, it gives an upper bound on the error and does not consider the correspondence between the two-point sets. We hope that the NEMNRR method, the way it is formulated, can provide (in the future) a way to compute the correspondence between those two sets of points, making HD error much more reliable.

### 6.2. Problem II: registration point distribution into FE-Mesh elements

One of the requirements we have yet to make much progress on is the *suboptimal distribution of the effective registration points,* i.e., once perceived outliers are rejected. This problem concerns mesh elements, and a very small number (same cases zero) of points makes the numerical formulation more sensitive to outliers and introduces additional displacement error due to integral voxel displacements recovered by block matching. The distribution of points also influences the condition of the [K + H^T^SH] matrix.

Over the last 15 years, we developed three different Image-to-Mesh (I2M) conversion methods for medical applications: (i) Body-Centered Cubic (BCC) was proposed by Molino et al. (33) and was implemented by Fedorov et al. (34) for a single-tissue and it was initially used in PBNRR (8). Then, a multi-tissue capability was used by Liu et al. (19, 20), and it was used in NEMNRR (13) and mesh gradation (i.e., control mesh size to reduce without compromising the fidelity of the mesh) by Drakopoulos et al. (35); (ii) Delaunay-based (27), and it is used in real-time ANRR with the results presented in Drakopoulos et al. (14, 24) and in Table 7; (iii) Lattice Decimation methods (36) because the relatively dense initial BCC mesh captures the object surface without much compression, thus preserving the good angles of the BCC triangulation. All three methods developed and evaluated for this project need further development regarding topologic accuracy in the presence of multi-tissue models. For example, one important question is: “How many materials can be accurately reconstructed around a mesh vertex or an edge so that the multi-tissue mesh is a topologically accurate representation of the input data?”

We have been working on yet another open question that involves generating meshes while considering the registration points recovered through the block-matching step. To our knowledge, no existing method in the literature addressed this question. Although we have made some progress, much work still needs to be done in this area. In Fedorov et al. (37), we attempted to improve the distribution of registration points over the mesh, using custom sizing functions for two different mesh generation methods (Delaunay refinement and Advancing Front). The evaluation was based on synthetic deformation fields and showed that the limited success of registration point equidistribution might reduce the registration error.

**Figure 6:**
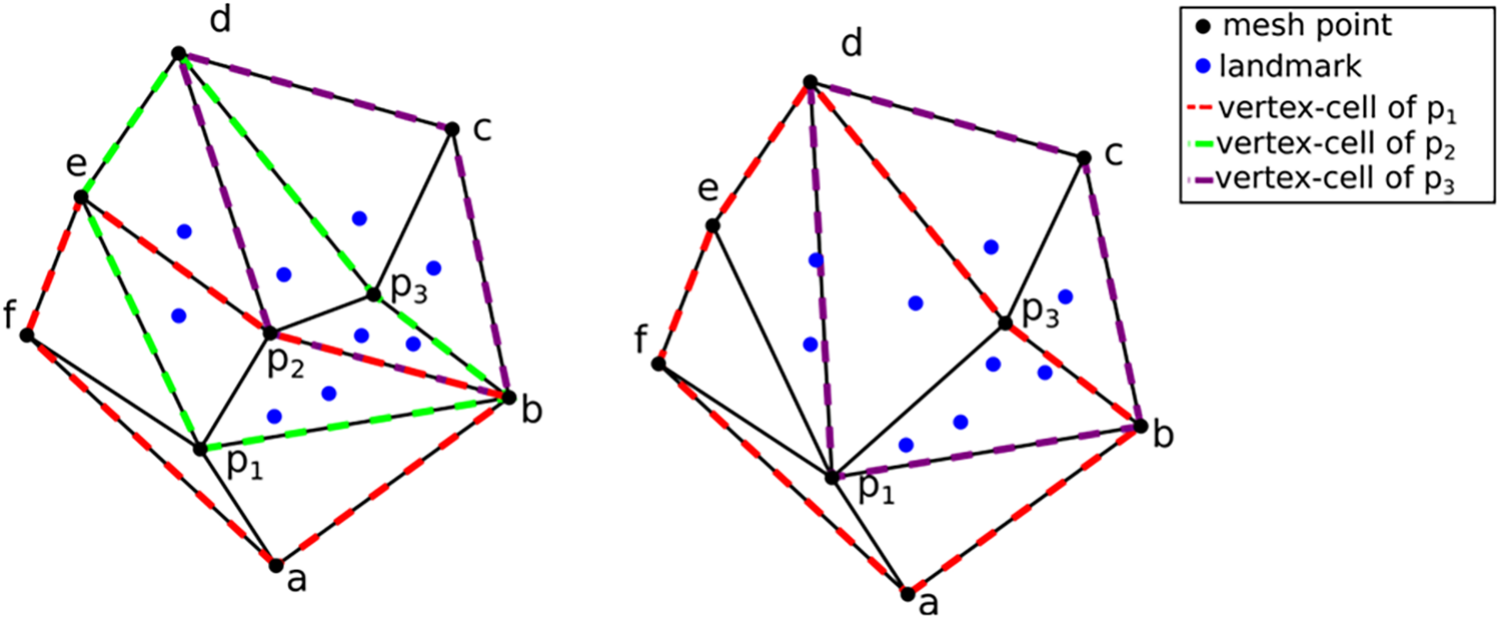
Optimizing landmark distribution.

For completeness, a summary of the method employed in Fedorov and Chrisochoides (29) is presented along with the modifications that can turn it into an anisotropic metric-based method. The (sub-)optimal distribution of the registration points can be formulated as assigning approximately the same number of registration points at each mesh vertex cell complex, where a mesh vertex cell complex is defined as the set of all the elements attached to a vertex. See, for example, Figure 6, left: the p1, p2, and p3 vertex cells on the left have 3, 7, and 5 landmarks, respectively. In Figure 6, right: by collapsing edge *p_2_p_1_*, one attempts to equidistribute the landmarks. Both the vertex cells of *p_1_* and *p_2_ now have* seven landmarks.

**Figure 7:**
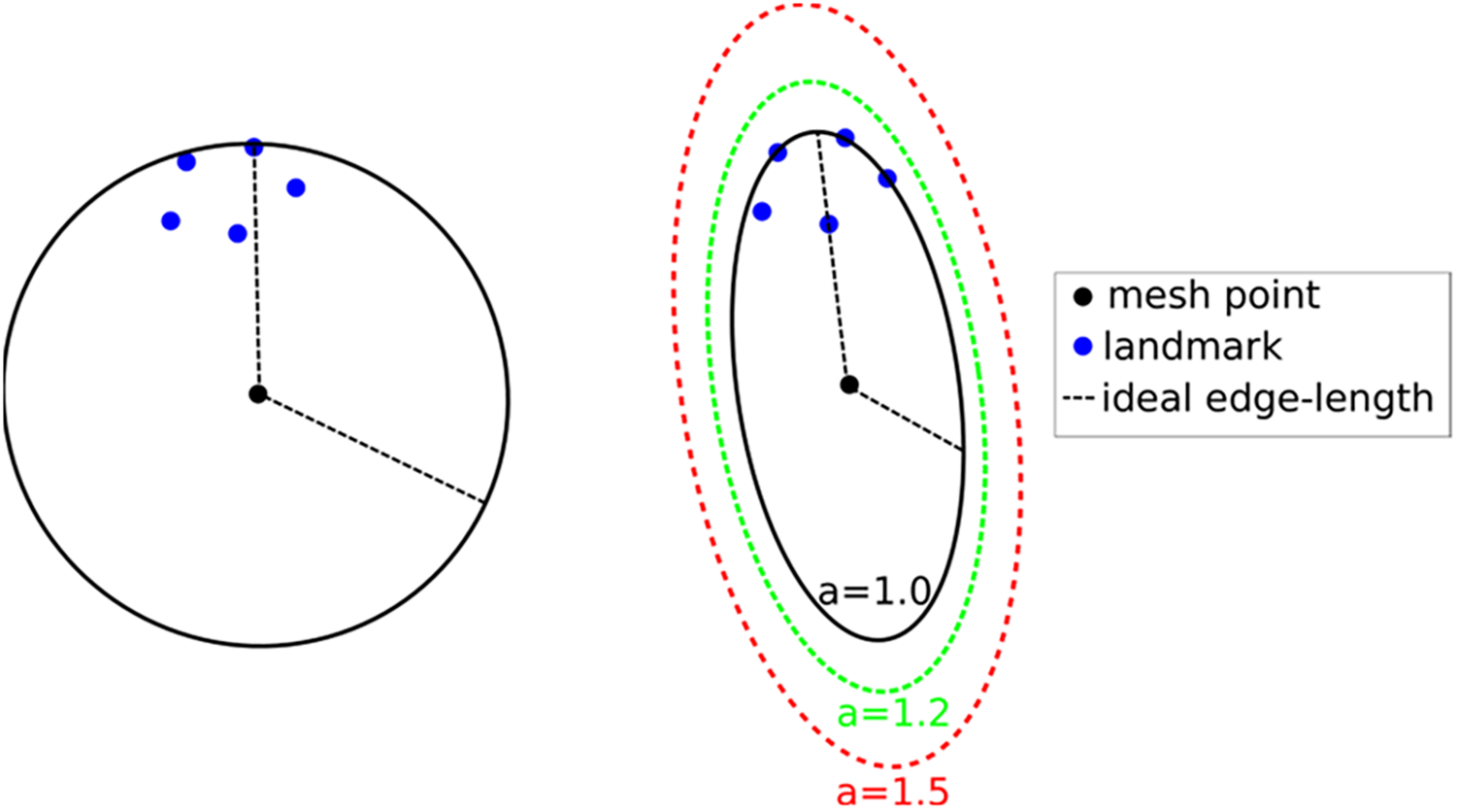
Left: isotropic metric that sets the spacing equal to the distance of the fifth closest registration point. Right: Anisotropic metric based on the five registration points for different values of the inflation constant. This Figure adapted from Drakopoulos et al. (24), Figure 5.

The crux of the method is to set the local spacing at each vertex equal to the distance to the k-th closest registration point. Assuming an ideal spacing, each vertex's mesh vertex cell complex will contain k registration points. An illustration for *k* = 5 is given in Figure 7 left. Notice that another way to interpret the sizing constraint at each vertex is using a sphere centered at each mesh vertex with a radius equal to the distance to the k-th registration point.

This technique produces adaptive meshes but does not efficiently capture the local distribution of landmarks. This is because only the k-th point is used, and the relative positions of the other k-1 landmarks are disregarded. To improve this, one can substitute the spheres at each vertex with the smallest bounding ellipsoid that encompasses the k closest registration points and is centered at the vertex. Describing the local spacing as an ellipsoid gives the ability to capture the local distribution of the landmarks better due to the increased degrees of freedom of an ellipsoid compared to a sphere (see, for example, Figure 7 right).

Creating the minimum volume ellipsoid that encloses a given pointset is a problem well studied in the convex optimization literature. The constructed ellipsoid has a natural mapping to a 3 × 3 positive definite matrix that can be used as a metric that guides the anisotropic mesh adaptation procedure. An additional flexibility to the mesh adaptation procedure can be introduced by an “inflation” (constant *a*), which is introduced and is common for all the points; it allows the enlargement of all ellipsoids by a constant factor. The goal of this parameter is to allow the mesh generation procedure to perform operations that may not conform to the strict size but improve the overall result. See Figure 7, right.

**Figure 8:**
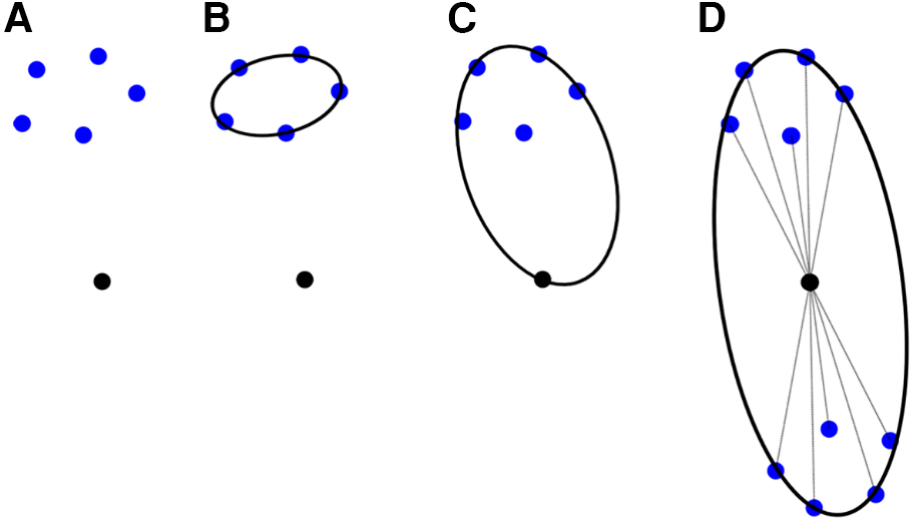
Different approaches to constructing a metric utilizing the minimum ellipsoid method.

**Figure 9:**
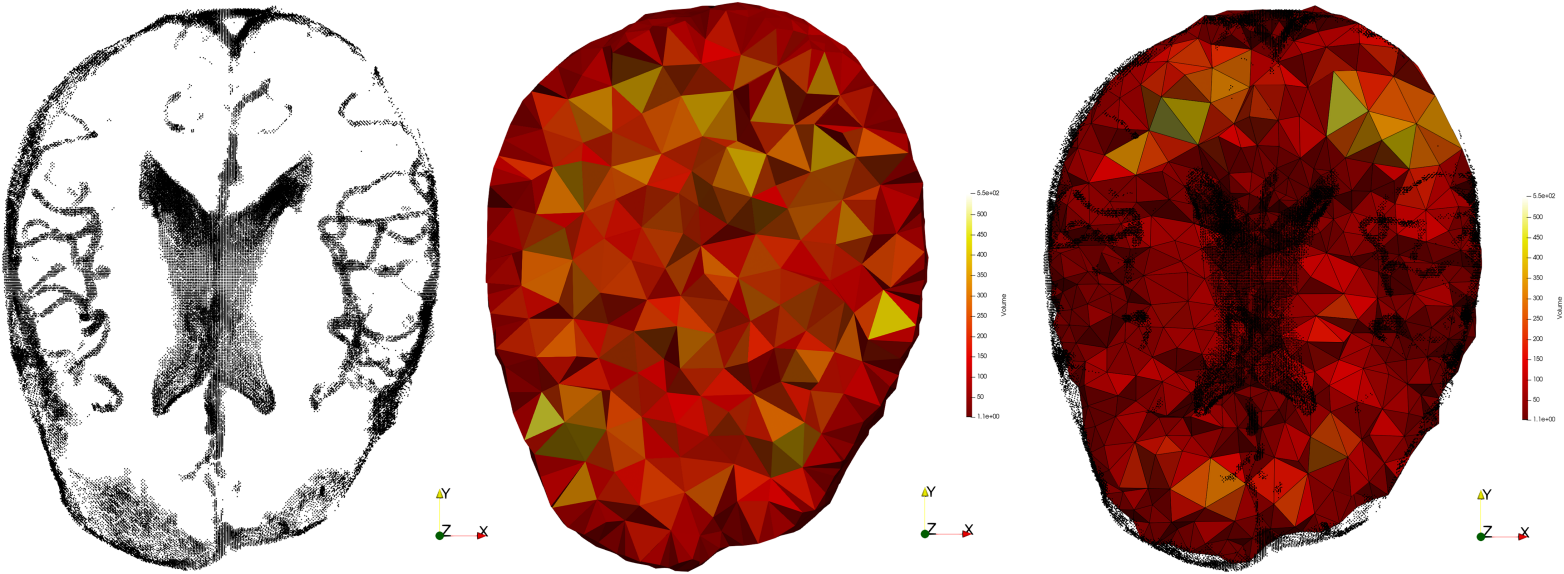
Registration points (left), isotropic mesh (center) and adaptive anisotropic sub-optimal mesh (right).

To incorporate the above approach to ANRR, the mesh generated by the Parallel Optimistic Delaunay Mesh (27) at each iteration, along with the landmarks identified by the Block-Matching step, are used to build a metric field. The metric field is constructed by iterating in parallel the mesh vertices and evaluating the k-closest registration points using a k-nn search from the VTK library (38). The minimum volume bounding ellipsoid is constructed using the Khachiyan algorithm. Directly using the landmarks (Figure 8B) will not yield an ellipse centered at a mesh point. Including the mesh point into the input of the minimum ellipsoid algorithm does not fix the issue (see Figure 8C). Instead, one can generate reflections of the k-closest landmarks by the mesh point and include them in the input of the minimum ellipsoid algorithm. Due to symmetry, the mesh point will always be in the center of the constructed ellipsoid. Finally, the mesh is adapted using MMG3D (39) using the metric field derived from the constructed ellipsoids. Figure 9 depicts the difference between isotropic and anisotropic sup-optimal mesh for a single case. Notice that the number of elements generated constrains the anisotropy; it must be approximately equal to the number of elements in the isotropic mesh.

Table 8 presents data from two cases: (A: case 9 from Drakopoulos et al. (24), provided by HSH (male, with glioma at Left Frontal location of the brain, where Partial Resection is performed, with preop-MRI and iMRI image sizes and spacing: 448 × 512 × 176 and 0.488 × 0.488 × 1.00, respectively) and (B: case 18 from Drakopoulos et al. (24), provided by HSH (female, with glioma at Left Frontal location of the brain, where Total Resection is performed, with preop-MRI and iMRI image sizes and spacing: 448 × 512 × 176 and 0.488 × 0.488 × 1.00, respectively).

**Table 8:** Hausdorff distance (HD) and error using landmarks by experts reported in mm.

	HD	Min error	Max error	Mean error	# tets	# vertices
Case A						
Baseline	2.24	1.07	5.90	3.51	13,210	3,264
Isotropic	1.95	1.22	7.53	3.71	19,893	4,177
Anisotropic (*a* = 1.0)	2.22	0.55	7.85	3.99	22,383	4.520
Anisotropic (*a* = 1.2)	2.00	1.01	7.10	3.70	17.593	3.629
Anisotropic (*a* = 1.5)	2.64	0.93	6.15	3.25	13,291	2,838
Case B						
Baseline	4.06	2.06	5.37	3.65	11,040	2,833
Isotropic	3.42	2.29	5.76	3.92	19,946	4,008
Anisotropic (*a* = 1.0)	3.71	2.12	5.50	3.96	22,342	4,460
Anisotropic (*a* = 1.2)	4.05	2.06	5.05	3.61	18,077	3.766
Anisotropic (*a* = 1.5)	4.05	1.92	5.17	3.65	13,812	2,983

Where baseline uses the default I2M within ANRR, isotropic uses the equidistribution method (29), and anisotropic uses different values for the alpha weight (in parenthesis).

From Drakopoulos et al. (24) and for case A, the HD error for Rigid Registration (RR) and PBNRR (without optimal distribution of registration points) is 10.59 mm and 10.76 mm, respectively. For case B, the HD error for RR and PBNRR (without optimal distribution of registration points) is 25.72 mm and 23.90 mm, respectively. In both cases, the sub-optimal distribution within the ANRR method reduced the error to about five to six times compared to RR and PBNRR. While the error using specific landmarks improved (see Table 7; see max and mean error columns), the expert evaluation indicates that more work is needed. So, this problem remains open and it needs to be considered in the context of point and element outlier rejection schemes presented in this paper.

## 7. Future work

While attempting to solve the combinatorial problems listed in Section 6 with classical computing, we plan to evaluate the use of Quantum Computing as well. Edge (or Feature) Detection kernel is the simplest to implement on Quantum Processing Unites (QPUs) and along with the Block Matching (together require about 15% of total execution time) is our next goal. The FEM-solver which accounts for 60% of the total time could utilize (in the future) a well-known quantum algorithm for linear systems of equations (41). In the preliminary results.

The Quantum Hadamard Edge Detection (QHED) is a quantum image processing algorithm that shows great promise, as demonstrated by Yao et al. (40). However, the circuit depth of the image encoding section of the algorithm becomes exponential with respect to the number of qubits required for encoding, which is a major drawback. In the future, with advancements in quantum sensing, it may be possible to eliminate this step altogether. For now, Noisy Intermediate-Scale Quantum (NISQ)-era one can manage exponential memory requirements, for large medical images (such as pre-op and intraoperative brain images) by using an image decomposition and processing in parallel the individual sub-images to address current qubit^4^ fidelity (or decoherence) issues.

**Figure 10:**
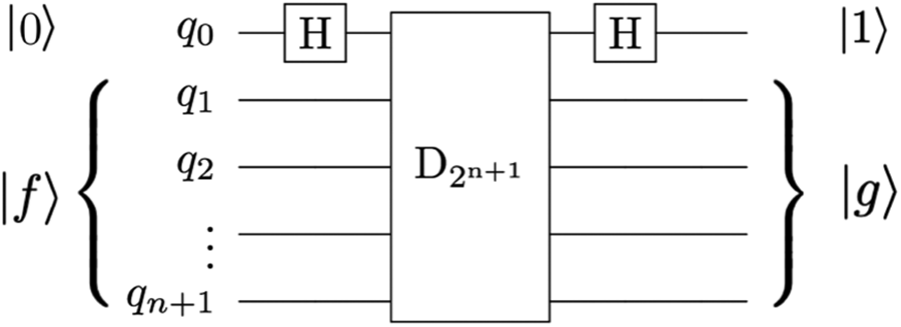
The QHED circuit proposed in Yao et al. (40) with an ancillary qubit. The D_2_^n + 1^ gate is a type of amplitude permutation that acts as a decrement operation on the input state vector.

The image decomposition scheme proposed by Yao et al. (40) can cause false edges that appear across the output image. In addition, the use of decrement permutation in the original QHED proposal, shown in Figure 10, requires a very large number of multi-controlled NOT (MCX) gates, which results in an polynomial (42) number of controlled not (CX) gates for the mapping of the QHED circuit onto the quantum computer hardware. In short, both the image encoding, and the edge detection parts of the algorithm produce exponential circuit depth, which compounds a massive loss in fidelity.

**Figure 11:**
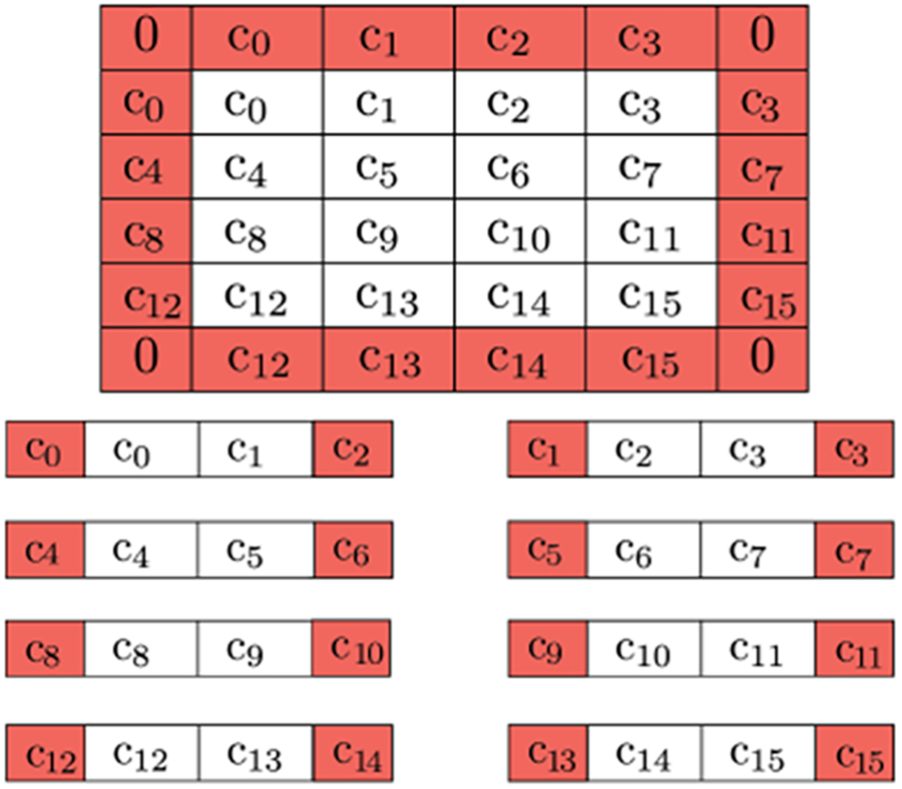
Buffer pixels of mirrored value are applied to the boundary of the overall image (0's for the corners, as they won’t be used). Further decomposition is possible by adding neighboring pixels to be used as a buffer due to QHED error. The red cells are ultimately disposed of in the final output.

To ensure correct boundary detection, we use classical space-filling curves commonly used in parallel numeric computations (43) to correct the artificial edges during the pre-and post-processing of input vectors. Figure 11 depicts our approach.

We use a linear number of ancillary qubits to reduce the number of CX gates. We also address fidelity concerns with optimization techniques from Ferris et al. (44) to minimize hardware noise in both the amplitude encoding and QHED circuits.

**Figure 12:**
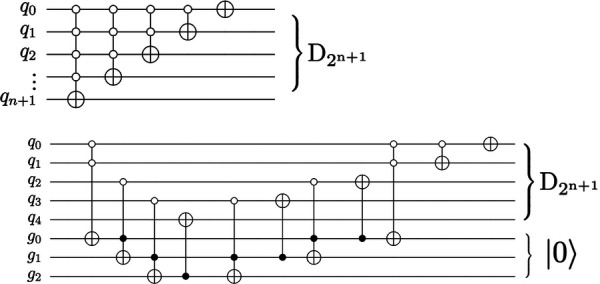
The circuit proposed in Yao et al. (40) for decrementing using *n* + 1 qubits require a descending series of MCX gates (top). An alternate decrement circuit (bottom) utilizes only CX and Toffoli gates, transpiling into a linear number of total CX gates.

**Figure 13:**
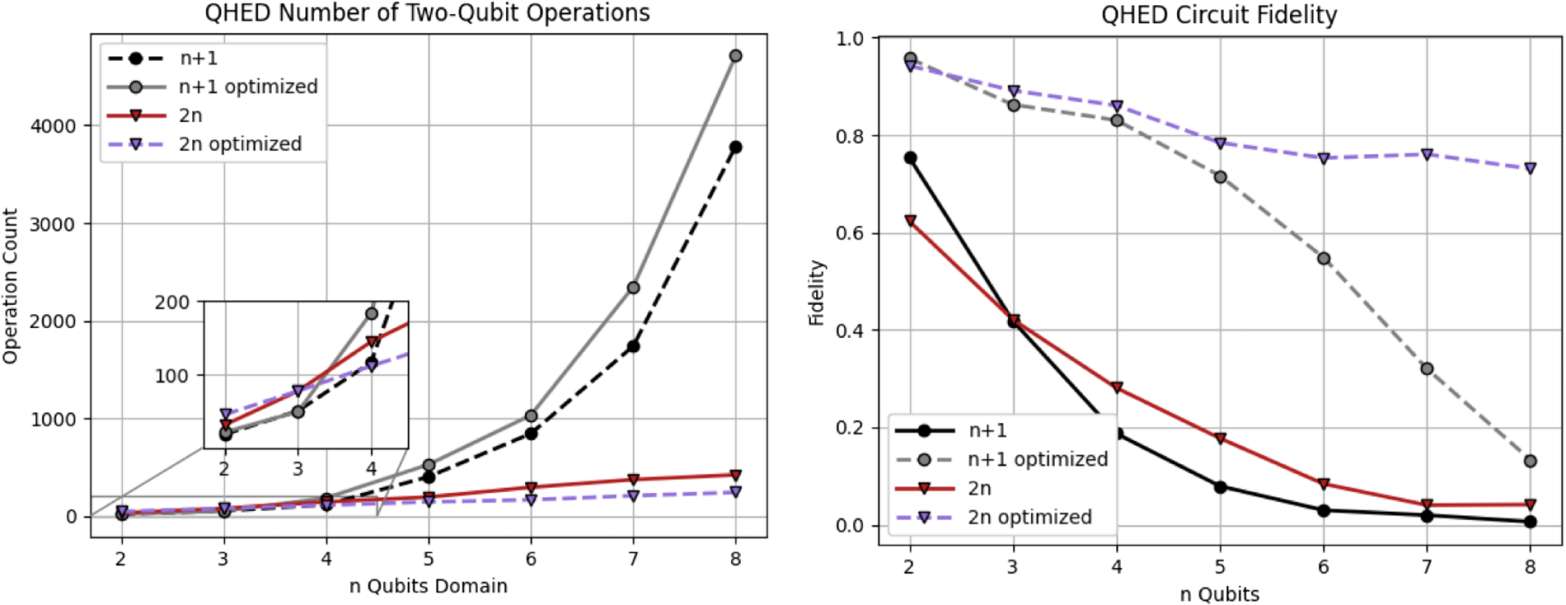
Analysis of the QHED algorithm using IBM's backend for both the proposed (red and blue) and Yao et al. (40) circuits (black and gray lines). Each of the two circuits is analyzed with and without full optimizations.

Optimizing the topology and software of quantum circuits can improve results on physical hardware. A simulated noisy backend from IBM (45) is utilized to evaluate the results of the proposed QHED optimizations (see Figure 12). Based on our analysis of Figure 13, even with the optimizations we made to the original circuit, we still need a polynomial amount of CX operations with respect to an increasing number *n* of data encoding qubits. This results in a rapid loss of fidelity for any *n *> 5 number of data encoding qubits.

In summary, we have improved the Quantum Edge Detection method (40) to generate comprehensible results on NISQ-era hardware for our use case. We introduced new: (1) pre-and post-processing classical steps by introducing space-filling curves and buffer pixels to eliminate image artifacts and (2) decrement permutation circuit, optimizations for realistic images on today's QPUs, and additional optimization techniques to improve circuit fidelity and reduce the depth and the number of two-qubit operations.

## 8. Conclusion

We compared three outlier rejection schemes for deformable registration of brain images. The PBNRR scheme, developed by Clatz et al., reduces the mean average error of state-of-the-art RR from 5.6 mm to 4.3 mm. This scheme rejects, on average, 24% of registration points as potential outliers and takes about a minute to complete for the cases we analyzed. The NEMNRR scheme, developed by Liu et al., extends the PBNRR method by considering both point and element outliers and improves the error by an additional 0.6 mm (to 3.7 mm). This scheme removes 28% of registration points as potential outliers and takes six times longer to complete the registration for the same cases. The ANRR scheme developed by Drakopoulos et al. relies on geometric means and the PBNRR method. This combination improves the RR error by an additional 1.1 mm (i.e., from 5.6 mm to 3.2 mm). We selected nine cases from two retrospective studies over the last decade, using evaluations completed by two independent groups of experts, one using 25 cases (13) and the other using 5 additional (30 total) cases (24).

The HD error analysis suggests that the outlier rejection schemes improve the average RR error (near the tumor resected area) from 12 mm: (i) to 8 mm for PBNRR, (ii) to 5 mm for NEMNRR, and (iii) 3.6 mm, for ANRR^5^. Based on an evaluation using the average max error metric from two groups of independent experts, it was found that the registration accuracy at specific landmarks improved from 8.9 mm with RR to 6.8 mm with PBNRR, 7.7 mm with NEMNRR, and 6.5 mm with ANRR. It is important to note that the accuracy of MRI data is dependent on both the resolution of the images and the specific acquisition protocol utilized (22). Varying voxel resolutions can lead to varying accuracy.

Combining outlier rejection schemes significantly improves registration accuracy using the HD metric. A byproduct of this work and specifically the even sub-optimal solution of Problems I and II, along with earlier work on the robust HD metrics (37) has the potential to enable reliable and automatic measurement of registration accuracy, which is vital for the development of iterative outlier rejection schemes. Finally, emerging technologies like Deep Learning, Cloud, and Quantum computing could be used to determine patient-specific parameters and better distribute and on-the-fly compute new registration points to help reduce element outliers. Preliminary results appeared (24) and Section 6 are promising. However, for this work to have a clinical impact, more work is required to improve the accuracy of cases with deep brain tumors and further validate the current state of the software.

## Footnotes

^1^Rigid registration implemented in 3D Slicer v4.4.0.

^2^B-Spline non-rigid registration implemented in 3D Slicer.

^3^PBNRR implemented in ITKv4.7.0.

^4^Qubit, like the bit in classical computers, is the unit of storing and processing information in quantum computing.

^5^Analysis from five additional cases indicates a further improvement from an average 19 mm HD error to 3.6 mm (22).

## Appendix I

**Table 9:** A few important parameters are used for PBNRR, NEMNRR, and ANRR.

Parameter	Value	Description
Initialization transform	Rigid	Rigid transformation to initialize the non-rigid registration
Connectivity pattern	“face”	Pattern for the selection of blocks
**F_s_**	5%	% selected blocks from total number of blocks
$B_{s,x}\times B_{s,y}\times B_{s,z}$	3 × 3 × 3	Block size (in voxels)
$W_{s,x}\times W_{s,y}\times W_{s,z}$	7 × 7 × 3 (BS)	Block matching window size (in voxels)
	9 × 9 × 3 (PR)	
	13 × 13 × 3 (TR, STR)	
**δ**	5	Mesh size (PBNRR, ANRR)
**E_b_**	2.1 KPA	Young's modulus for brain parenchyma
**E_t_**	21 KPA	Young's modulus for tumor
**v_b_**	0.45	Poisson ratio for brain tumor and parenchyma
**E_t_**	0.1	Poisson ratio for ventricle (NEMNRR)
**F_r_**	25%	% of rejected outlier blocks
**N_rej_**	10	Number of outlier rejection steps
**N_iter, max_**	10	Max number of adaptive iterations (ANRR)

**Table 10:** Parameters used in this study for rigid registration (RR) and B-spline non-rigid registration methods implemented in 3D slicer.

Interpolation mode	Linear	Linear	
Sampling percentage	5%	5%	Percentage of image voxels sampled for MMI
Histogram bins	100	100	Number of histogram levels
Optimizer type	VR3DT	LBFGSB	–
Max number of iterations	1,500	1,500	Maximum number of iterations for optimizer
Grid size	–	15 × 15 × 15	Number of subdivisions of the B-Spline grid
Min step length	10^−3^	10^−3^	Min threshold step for optimizer
Projected gradient tolerance	–	10^−5^	Used by LBFGSB

MMI, mattes mutual information; VR3DT, versor rigid 3D transform; LBFGSB, limited memory broyden fletcher goldfarb shannon minimization with simple bounds.
